# Supplementary figures and images for: Generation of Tailored Extracellular Matrix Hydrogels for the Study of In Vitro Folliculogenesis in Response to Matrisome-Dependent Biochemical Cues
Source: Bioengineering (Basel). 2024 May 25;11(6):543. doi: 10.3390/bioengineering11060543 (PMC11200611; doi:10.3390/bioengineering11060543)

## Supplementary Material:

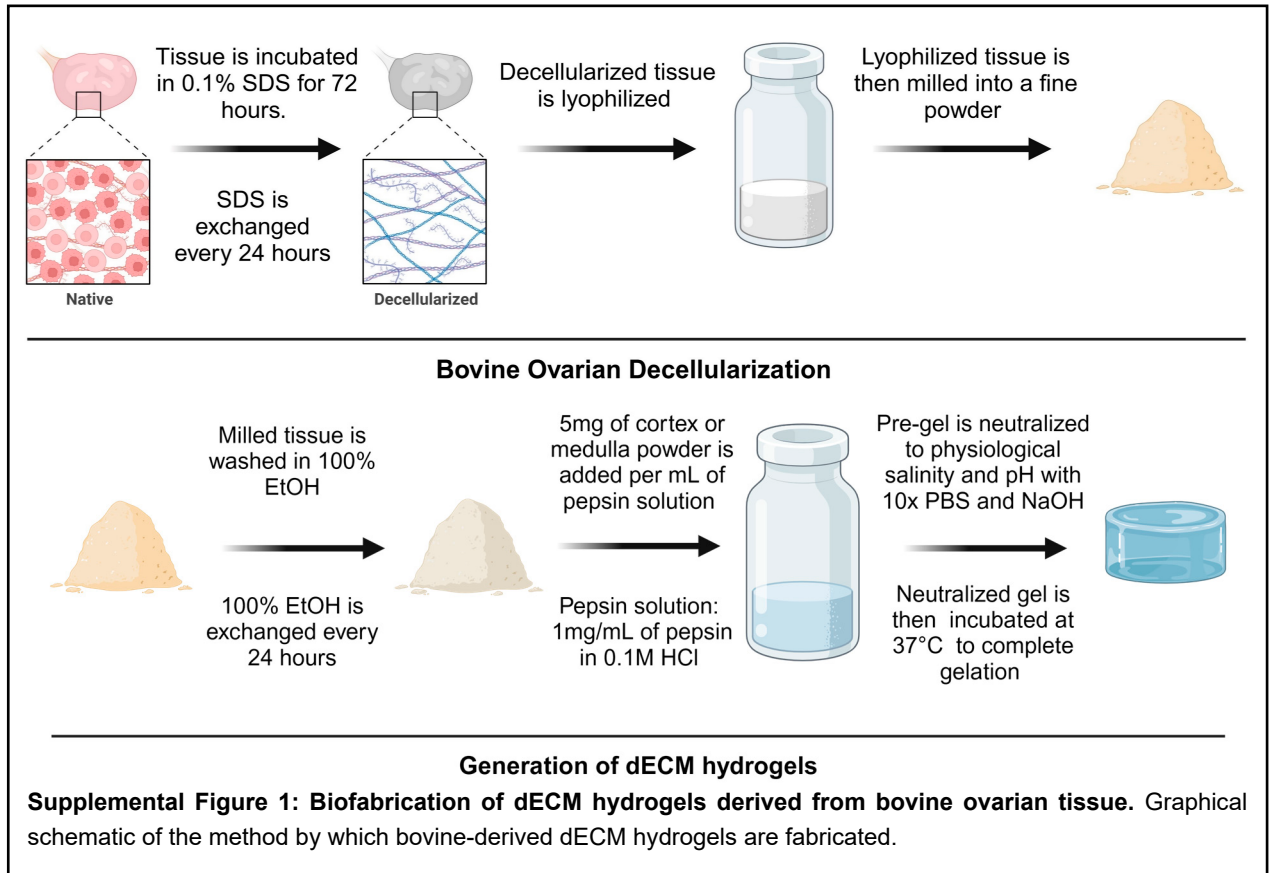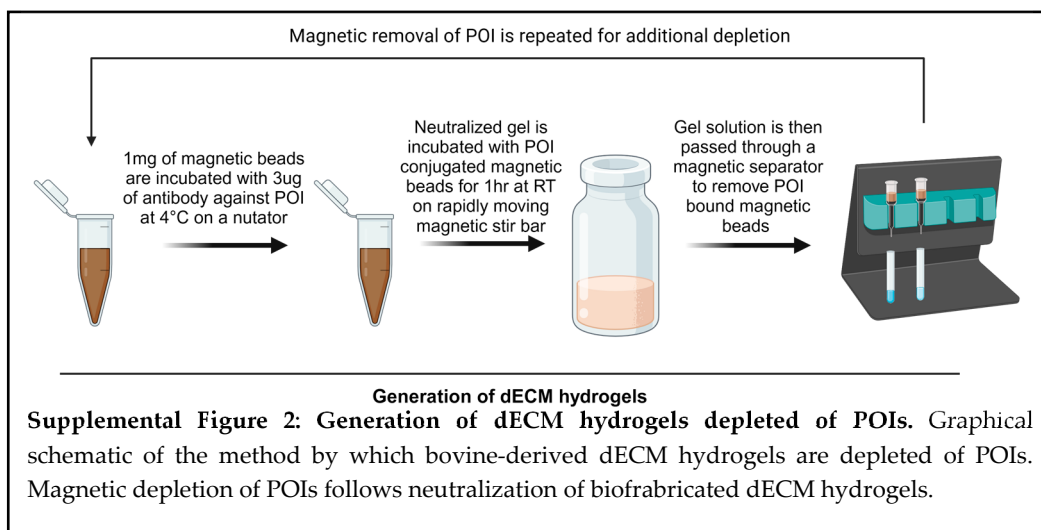

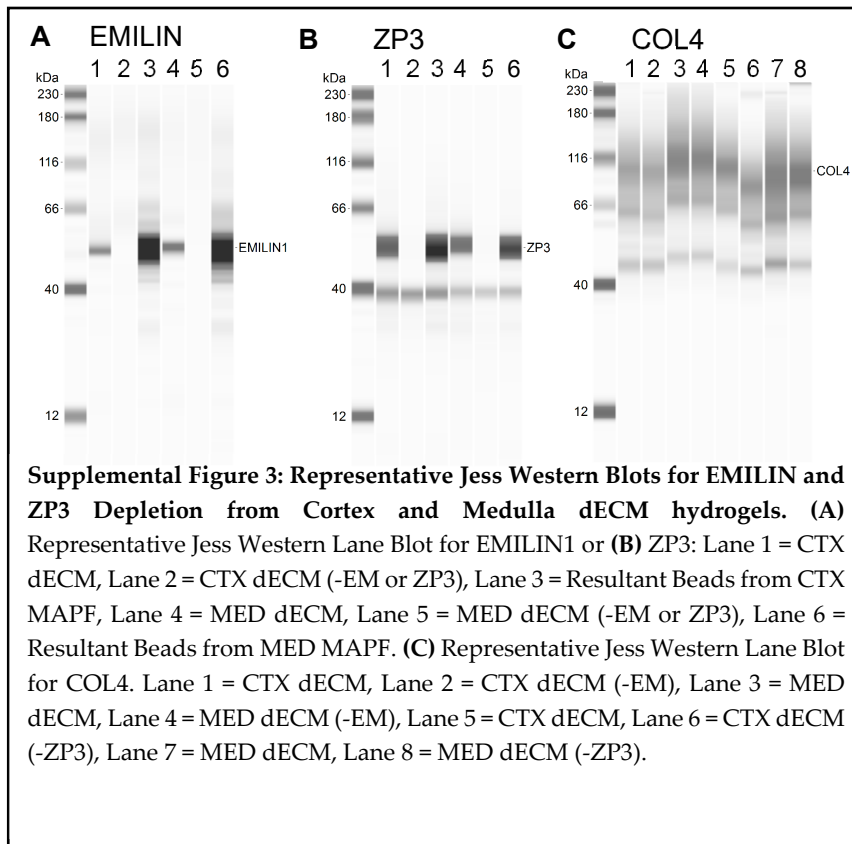

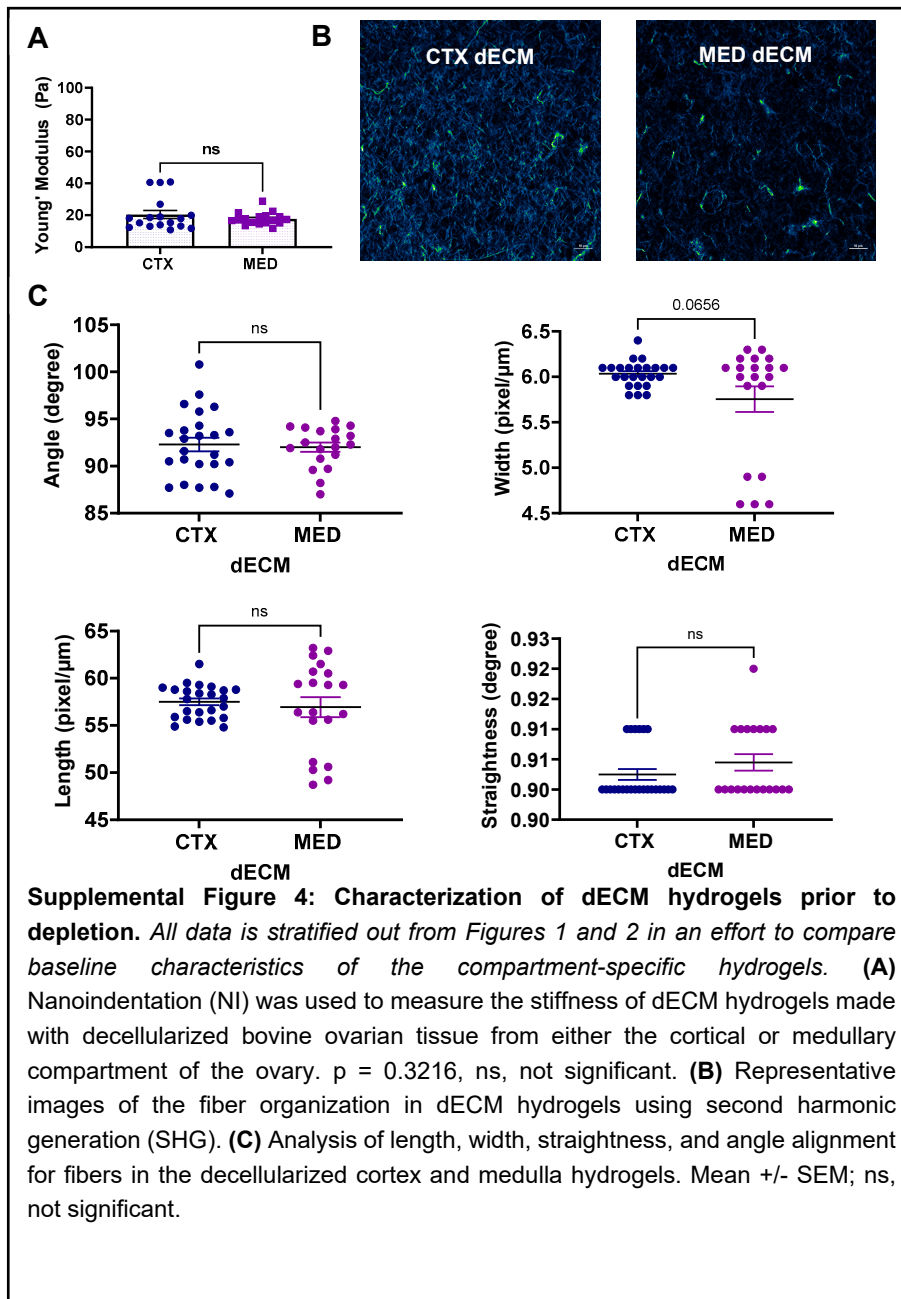

Supplement: Supplementary file 1 [file bioengineering-11-00543-s001.zip › bioengineering-3017743-supplementary.pdf]
